# The Cholinergic Lateral Line Efferent Synapse: Structural, Functional and Molecular Similarities With Those of the Cochlea

**DOI:** 10.3389/fncel.2021.765083

**Published:** 2021-10-12

**Authors:** Paola V. Plazas, Ana Belén Elgoyhen

**Affiliations:** ^1^Instituto de Farmacología, Facultad de Medicina, Universidad de Buenos Aires, Buenos Aires, Argentina; ^2^Instituto de Investigaciones en Ingeniería Genética y Biología Molecular, Consejo Nacional de Investigaciones Científicas y Técnicas, Buenos Aires, Argentina

**Keywords:** α9α10, efferent, cochlea, lateral line, nicotinic receptor, zebrafish

## Abstract

Vertebrate hair cell (HC) systems are innervated by efferent fibers that modulate their response to external stimuli. In mammals, the best studied efferent-HC synapse, the cholinergic medial olivocochlear (MOC) efferent system, makes direct synaptic contacts with HCs. The net effect of MOC activity is to hyperpolarize HCs through the activation of α9α10 nicotinic cholinergic receptors (nAChRs) and the subsequent activation of Ca^2+^-dependent SK2 potassium channels. A serious obstacle in research on many mammalian sensory systems in their native context is that their constituent neurons are difficult to access even in newborn animals, hampering circuit observation, mapping, or controlled manipulation. By contrast, fishes and amphibians have a superficial and accessible mechanosensory system, the lateral line (LL), which circumvents many of these problems. LL responsiveness is modulated by efferent neurons which aid to distinguish between external and self-generated stimuli. One component of the LL efferent system is cholinergic and its activation inhibits LL afferent activity, similar to what has been described for MOC efferents. The zebrafish (*Danio rerio*) has emerged as a powerful model system for studying human hearing and balance disorders, since LL HC are structurally and functionally analogous to cochlear HCs, but are optically and pharmacologically accessible within an intact specimen. Complementing mammalian studies, zebrafish have been used to gain significant insights into many facets of HC biology, including mechanotransduction and synaptic physiology as well as mechanisms of both hereditary and acquired HC dysfunction. With the rise of the zebrafish LL as a model in which to study auditory system function and disease, there has been an increased interest in studying its efferent system and evaluate the similarity between mammalian and piscine efferent synapses. Advances derived from studies in zebrafish include understanding the effect of the LL efferent system on HC and afferent activity, and revealing that an α9-containing nAChR, functionally coupled to SK channels, operates at the LL efferent synapse. In this review, we discuss the tools and findings of these recent investigations into zebrafish efferent-HC synapse, their commonalities with the mammalian counterpart and discuss several emerging areas for future studies.

## Introduction

The acquisition and processing of external stimuli are essential for all life forms to react appropriately to environmental cues. Sensory systems acquire information from the surrounding world employing specialized receptor cells at the periphery. They translate those stimuli into electrical signals that are then decoded by the central nervous system (CNS). In the mammalian auditory system, sound detection begins when sound waves strike the eardrum, which transmits that vibrational stimulus to the organ of Corti, the sensory epithelium of the mammalian inner ear. There, the inner hair cells (IHCs) transform mechanical input into electrical signals that are sent to the CNS by the auditory nerve (Hudspeth, 1997a). However, unlike vision, touch and the chemical senses, sound transduction is modulated at the periphery by efferent signals that descend from the brain to the inner ear (Guinan and Stankovic, 1996).

Most of what we know concerning efferent/hair-cell (HC) physiology has been built by studies in rodents. In mammals, two types of cochlear HCs are arranged in rows along the organ of Corti. Inner hair cells (IHCs) are the primary receptor cells and receive nearly all the afferent innervation. Outer hair cells (OHCs) are involved in sound amplification and fine tuning of the basilar membrane (Hudspeth, 1997b). They are the target of an efferent neural pathway, the medial olivocochlear (MOC) fibers, that makes direct contact at the base of the OHCs (Rasmussen, 1946; Guinan et al., 1983; Guinan and Stankovic, 1996; Warr et al., 1997). IHCs are also the target of efferent fibers, the lateral olivocochlear pathway, but in this case the efferent terminals make axo-dendritic contacts with auditory afferent fibers. Within the mammalian inner ear the net effect of MOC activity is to hyperpolarize OHCs (Guinan and Stankovic, 1996). Although the inhibitory signature of the efferent synapses to inner-ear HCs, by hyperpolarization, was established early on by Housley and Ashmore (1991) and Fuchs and Murrow (1992b), how this process is brought about at the molecular level remained unclear until the cloning of the genes encoding the α9 and α10 cholinergic nicotinic receptor (nAChR) subunits (Elgoyhen et al., 1994, 2001). Since then, it has been established that the receptor that mediates neurotransmission at the MOC-HC synapse is a pentameric α9α10 nAChR with very peculiar functional properties and high calcium (Ca^2+^) permeability (Elgoyhen et al., 2001; Weisstaub et al., 2002; Gómez-Casati et al., 2005; Elgoyhen and Katz, 2012; Lipovsek et al., 2012, 2014). ACh-gated depolarization is followed by activation of Ca^2+^-dependent SK2 potassium (K^∗^) channels and subsequent OHC hyperpolarization (Dulon et al., 1998).

A serious obstacle in research on many mammalian sensory systems in their native context is that their constituents are difficult to access even in newborn animals. That is the case of the inner ear, which is encased in bone, thus making it impracticable to study circuit assembly, mapping, or controlled manipulation in its native environment. By contrast, fishes and amphibians have a superficial and accessible mechanosensory system, the lateral line (LL), which circumvents many of these problems. LL responsiveness is modulated by efferent neurons which aid to distinguish between external and self-generated stimuli (Pichler and Lagnado, 2020). One component of the LL efferent system is cholinergic and its activation inhibits LL afferent activity (Russell, 1971b; Russell and Roberts, 1972; Flock and Russell, 1973a, 1976; Lunsford et al., 2019; Pichler and Lagnado, 2020), similar to what has been described for MOC efferents.

The zebrafish (*Danio rerio*) has emerged as a powerful model system for studying human hearing and balance disorders since LL HCs are structurally and functionally analogous to cochlear HCs (Whitfield, 2002; Nicolson, 2005), but are optically and pharmacologically accessible within an intact specimen, facilitating high-resolution *in toto* live imaging. In addition, the genetic toolbox for zebrafish, combined with pharmacological and optogenetic approaches enable well-controlled manipulations of neurons in their natural context with spatiotemporal precision, providing a powerful paradigm to study the assembly of the neural circuits that underlie the central representation of spatially heterogeneous hydromechanic stimuli and their behavioral output (Ghysen et al., 2007).

Complementing mammalian studies, zebrafish have been used to gain significant insights into many facets of HC biology, including mechanotransduction and synaptic physiology (Kindt and Sheets, 2018), as well as mechanisms of both hereditary and acquired HC dysfunction (Nicolson, 2005; Sheets et al., 2021). However, with the rise of the zebrafish LL as a model in which to study auditory system function and disease, there has been an increased interest in studying its efferent system and evaluate the similarity between mammalian and piscine efferent synapses.

The present work reviews data which has helped advance our understanding of how the zebrafish efferent-HC synapse operates and discuss their commonalities with the mammalian counterpart.

## The Zebrafish Lateral Line

**FIGURE 1:**
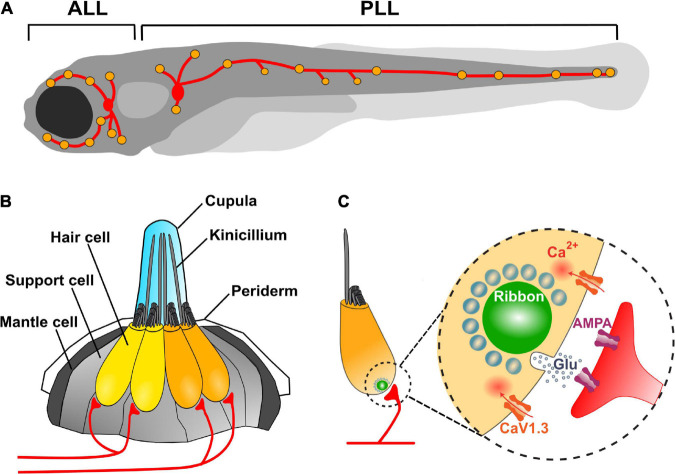
Zebrafish lateral line neuromasts and their afferent innervation. **(A)** Schematic depicts a larval zebrafish. Yellow patches indicate the location of neuromasts in the LL system. Red patches represent the location of the anterior (ALL) and posterior (PLL) LL ganglia. The neurons in these ganglia project to and innervate HCs in the LL. **(B)** A side view of a single LL neuromast. HC (yellow and orange) are surrounded by supporting cells (light gray) and innervated by both afferent (red) and efferent neurons (not shown). Mantle cells (dark gray) outline the neuromast and contact the periderm cells of the larva’s integument. Apical mechanosensory hair bundles and kinocilia are contained within a gelatinous cupula and project out into the water. A neuromast contains two populations of HCs of opposing hair bundle polarities (yellow and orange). Each afferent neuron (red) exclusively synapses with HCs of identical planar orientation. **(C)** Diagram of a single HC. HCs are activated when hair bundles are deflected and mechanosensitive channels open allowing the influx of cations. This apical activity depolarizes the HC, resulting in presynaptic Ca^2+^ influx and release of glutamate onto the afferent neuron. A presynaptic density called a ribbon (green) helps to recruit synaptic vesicles (light blue circles) to the synapse near clusters of CaV1.3 channels.

Fishes and amphibians have a mechanosensory system, the LL, which detects local water currents and mediates a large variety of behaviors, from prey detection to predator avoidance, schooling and rheotaxis (Partridge and Pitcher, 1980; Bleckmann and Zelick, 2009; McHenry et al., 2009; Olszewski et al., 2012; Suli et al., 2012; Olive et al., 2016; Oteiza et al., 2017). The LL comprises a set of sensory organs, the neuromasts, composed of a core of mechanosensitive HCs surrounded by non-sensory cells (Figures 1A,B). HCs are innervated by afferent and efferent neurons (Metcalfe et al., 1985; Raible and Kruse, 2000). Neuromast are arranged in series along the fish body and head and located superficially just beneath the fish skin, with their apical hair bundles and kinocilia, enclosed in a gelatinous cupula, protruding into the aqueous environment. Neuromasts on the head form the anterior lateral line (ALL), while those on the trunk and tail form the posterior lateral line (PLL) (Figure 1A). The cupula encases the filaments, granting them stiffness and protection (McHenry and Van Netten, 2007; McHenry et al., 2008). Furthermore, since the hair bundle resembles a lever pivoting on a fulcrum, the physical properties of the cupula, such as length and stiffness, might determine the sensitivity of each HC (Van Trump and McHenry, 2008).

## Zebrafish Hair Cells

Zebrafish HCs are remarkably similar to mammalian HCs at the molecular and cellular level (Whitfield, 2002; Coffin et al., 2004; Nicolson, 2005). Genetic studies have demonstrated that numerous genes required for hearing and balance in zebrafish are also required in mice and humans (Coffin et al., 2004; Nicolson, 2005; Varshney et al., 2016). Like in all HC-containing sensory organs, LL HC function involves two distinct processes: mechanoelectrical transduction and electrochemical transduction. These functions are critically dependent on two subcellular specializations: the hair bundle and the ribbon synapse. The hair bundle is notable for its exquisite sensitivity to mechanical stimuli, which it translates into membrane depolarizations (Hudspeth and Corey, 1977). The ribbon synapse bears the arduous task of continuously reporting the membrane voltage in a temporally precise fashion through the release of glutamate onto postsynaptic afferent neurons (Keen and Hudspeth, 2006).

Hair cells transduce mechanical force (generated by moving fluids) into electrical signals. by means of their apical “hairs” that protrude out of the cell into its immediate environment (Hudspeth, 1989). Deflections of the hair bundles open cation-permeable channels, resulting in receptor potentials that can lead to vesicle release from the cell’s basal surface at presynaptic specializations known as synaptic ribbons (Corey and Hudspeth, 1979; Keen and Hudspeth, 2006). L-type voltage-gated Ca^2+^ channels (CaV1.3) positioned in the basolateral membrane, adjacent to synaptic ribbons, mediate the influx of Ca^2+^ during membrane depolarizations (Sidi et al., 2004). The proximity of CaV1.3 channels to the sites of vesicle fusion facilitates the Ca^2+^-dependent release of glutamate into the synaptic cleft (Brandt et al., 2005; Figure 1C). Importantly, HCs are directionally sensitive, which means that they become maximally depolarized when stimulated in a preferred direction and hyperpolarized when stimulated against the preferred direction (Hudspeth and Corey, 1977). In general, the magnitude of the electrical response is graded with respect to the magnitude and not with the velocity of deflection (Flock, 1965).

Patch clamp analysis of zebrafish HCs has allowed comparison of the physiological properties between HCs from different locations (LL vs. inner ear) and with mammals (Ricci et al., 2013; Olt et al., 2014, 2016). Zebrafish HCs physiologically resembled those of other lower vertebrates, and to some extent, the HCs from immature mammalian vestibular and auditory systems (Olt et al., 2014).

### Planar Polarization of Lateral Line Hair Cells

A hallmark of LL HCs is that, within a neuromast, each cell exhibits an inherent polarity. On one hand, directional preference is determined by the morphology of the hair bundle itself. In the LL, each bundle is formed by a staircase of actin-based stereocilia and a microtubule-based kinocilium eccentrically located adjacent to the tallest stereocilia, and connected to each other via tip-links (Pickles et al., 1984; Kindt et al., 2012). This structural asymmetry confers direction selectivity to the HC: forces that deflect the bundle toward the kinocilium, become excitatory by stretching the tip-links and providing the force necessary to open the mechanosensory channels (Pickles et al., 1984; Kindt et al., 2012). Conversely, deflections away from the kinocilium result in hyperpolarization. Moreover, the stimulus coding is non-linear, since deflections in the preferred direction elicit currents that are larger in magnitude than the currents caused by equal deflections in the non-preferred orientation (Flock and Wersall, 1962). However, in the LL, further directional organization is imposed at the anatomical level given that neuromast HCs are not positioned in random directions. Rather, each neuromast contains only two populations of HCs of opposing polarities along either the rostro-caudal or dorso-ventral axis of the fish (Flock and Wersall, 1962). Consequently, half of the HCs respond to stimuli from one direction (i.e., deflection toward the posterior) and the other half respond to stimuli from the other (deflection toward the anterior) (Rouse and Pickles, 1991; López-Schier et al., 2004; Ghysen et al., 2007).

## Neuromast Afferent Innervation

At their basal surface, LL HCs exhibit on average three ribbon synapses that contain specialized electron-dense presynaptic structures, known as synaptic ribbons, that tether and stabilize glutamatergic synaptic vesicles at the active zone, near presynaptic clusters of CaV1.3 channels (Frank et al., 2010; Sheets et al., 2011, 2012). As a consequence of deflection in the appropriate direction, HCs release glutamate at ribbon-synapses in close apposition to the terminals of afferent neurons (Obholzer et al., 2008). The soma of these primary sensory neurons are located in small cranial ganglia. Afferent neurons that innervate head neuromasts are located in the ALL ganglion, found posterior to the eyes, and those that innervate tail and dorsal neuromasts are located in the PLL ganglion, posterior to the ears (Corey and Hudspeth, 1979; Raible and Kruse, 2000; Keen and Hudspeth, 2006; Figure 1A). Single afferent neurons may innervate more than one neuromast but, within a neuromast, they form bouton-like synapses exclusively on nearly all HCs of the same polarity (Figure 1B; Nagiel et al., 2008; Obholzer et al., 2008; Faucherre et al., 2009; Sheets et al., 2011; Pujol-Martí et al., 2014).

Notably, afferent neurons only collect information from HCs on the ipsilateral side with respect to their somas, giving rise to a mirror-symmetric circuit through the fish’s midline. Physiological responses of primary sensory neurons are phase-locked to deflections of the HCs they innervate (Trapani et al., 2009; Haehnel et al., 2012). In the absence of external stimuli, sensory neurons display spontaneous activity that is generated by basal spontaneous HC glutamate release (Trapani et al., 2009; Trapani and Nicolson, 2011). Spontaneous spiking in sensory neurons is thought to be critical for information coding (Douglass et al., 1993). Neurons with an elevated resting state have a greater dynamic range to code for both increases and decreases of their inputs. This is extremely relevant for LL sensory neurons that innervate HCs, which can be depolarized or hyperpolarized depending on the direction of the mechanosensory stimulus.

Afferents from both the ALL and PLL project central axons to contact second-order output neurons located in the ipsilateral medial octavolateralis nucleus (MON) of the hindbrain (Metcalfe et al., 1985; Liao, 2010), forming a somatotopic map, whereby dorsal axons correspond to more posterior neuromasts (Alexandre and Ghysen, 1999). A limited number of afferents also converge close to the lateral dendrite of the Mauthner cell, a command neuron that triggers quick escapes, consistent with the observation that the LL can mediate escape behaviors (Kimmel et al., 1974; Eaton et al., 1977; Haehnel et al., 2012; Pujol-Marti et al., 2012). Given that sensory neurons terminate in at least two distinct regions in the hindbrain, it has been suggested the existence of two functionally segregated sensory streams (Liao, 2010; Haehnel et al., 2012; Pujol-Marti et al., 2012). One is composed of large early-born afferent neurons that can innervate multiple neuromasts, and whose axonal projections converge onto medial regions of the hindbrain in proximity to the Mauthner cell. The second stream is made up of smaller, later-born neurons that innervate single neuromasts and project to the MON. In this case, neurons possess more limited receptive fields and heightened sensitivity, which would be useful to detect and localize subtler inhomogeneities in the water surrounding the animal.

Experiments using anterograde transport of dyes have identified higher order neurons in the ipsi- and contralateral hindbrain, the optic tectum and the torus semicircularis, which is equivalent to the mammalian inferior colliculus, the major target of auditory information (Barrett, 1973; Wubbels et al., 1997; Bleckmann, 2008). In agreement with these anatomical studies, functional experiments using calcium imaging have shown different non-overlapping activation patterns in the tectum when fish are presented with water flow, auditive or visual stimuli (Thompson et al., 2016).

### Functional Heterogeneity and Redundancy

Striking features of the LL system are its anatomical redundancy and functional heterogeneity, which highlight the complexity underlying LL HC function *in vivo*.

Anatomical redundancy has been described at three levels. First, each of the two HC populations (responding to either an anterior or posterior directed stimulus) (Jian et al., 2017) is represented by multiple HCs. Second, each HC has approximately three afferent synapses per HC (Sheets et al., 2012) and third, each HC is innervated by more than one afferent neuron (Pujol-Martí et al., 2014). In addition, functional heterogeneity among HCs within the same neuromast has been shown since, in their native environment, all HCs are mechanosensitive but the majority of them are synaptically silent (Zhang et al., 2018). It has been proposed that anatomical redundancy and presynaptic silencing may work together to prevent unnecessary energy loss and cellular stress, and to rapidly backup cells lost after damage. In addition, LL afferent neurons display heterogeneous anatomical and physiological properties that delineate a dimorphic afferent neural map (Liao and Haehnel, 2012; Pujol-Marti et al., 2012).

## Neuromast Efferent Innervation

Vertebrate HC systems, including the cochlea, vestibular and LL organs are innervated by descending efferent fibers that modulate their response to external stimuli (Russell, 1971b; Metcalfe et al., 1985; Guinan and Stankovic, 1996; Bricaud et al., 2001). In the LL, the excitation of efferent fibers inhibits afferent activity by generating inhibitory postsynaptic potentials in HCs (Russell, 1971b; Flock and Russell, 1973a, 1976). In addition, excitatory efferent effects can be observed when cholinergic transmission is blocked (Russell, 1971a; Flock and Russell, 1973b).

**FIGURE 2:**
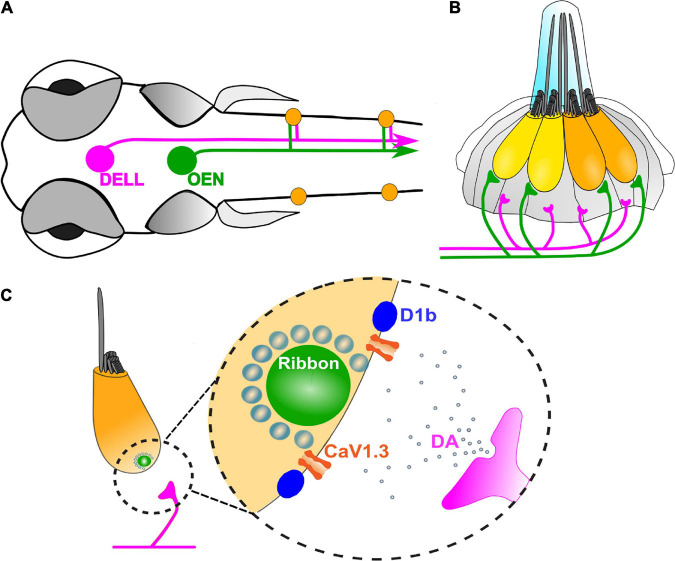
Zebrafish efferent system. **(A)** Schematic representation of the head of a zebrafish larva, with the three efferent nuclei: the Diencephalic Efferent of the Lateral Line (DELL, magenta), and the Rostral and Caudal Efferent Nuclei, collectively referred to as the Octavolateral Efferent Nucleus (OEN, green). Neuromasts (orange) are innervated by descending inputs that originate from the DELL in the ventral hypothalamus (magenta) and from the OEN in the hindbrain (green) (Hashimoto et al., 1970; Russell and Roberts, 1972; Zottoli and Van Horne, 1983; Metcalfe et al., 1985; Tricas and Highstein, 1991; Bricaud et al., 2001). The circuit is midline symmetric but only one side is illustrated for clarity. **(B)** A side view of a single LL neuromast. One OEN fiber contacts the base of all HCs within a neuromast, independently of their polarities (Dow et al., 2018). DELL terminals are found beneath the HC layer, where supporting cells reside, but not in close apposition to HCs (Toro et al., 2015). **(C)** Paracrine signaling by DA is mediated by D1b receptors which are tightly localized to presynaptic zones of HCs, directly adjacent to synaptic ribbons. Activation of D1b receptors leads to an increase in the activity of presynaptic Cav1.3a channels, thus inducing a stimulatory effect on HC activity (Toro et al., 2015).

Anatomical studies in fishes revealed that LL efferent neurons locate their somas in three nuclei in the CNS (Hashimoto et al., 1970; Russell and Roberts, 1972; Zottoli and Van Horne, 1983; Metcalfe et al., 1985; Tricas and Highstein, 1991; Bricaud et al., 2001). The rostral and caudal efferent nuclei located in the hindbrain are cholinergic, and are collectively referred to as the octavolateral efferent nucleus (OEN). The third nucleus, the diencephalic efferent of the LL (DELL) is catecholaminergic, and is found in the ventral hypothalamus (Bricaud et al., 2001; Tay et al., 2011; Figure 2A). In addition, efferent neurons that innervate the LL and ear of the eel produce both acetylcholine and calcitonin gene-related peptide (CGRP), suggesting that the already identified efferent nuclei could account for multiple transmitters (Roberts et al., 1994).

Dopaminergic efferent terminals are located within the supporting cell layer but do not directly contact LL HCs (Figure 2B). However, D1b receptors that are tightly localized to ribbon synapses, mediate synaptic transmission at the dopaminergic LL efferent synapse (Figure 2C; Toro et al., 2015). This is in contrast to the expression pattern of D1R reported for the organ of Corti (Maison et al., 2012) and rodent vestibular organs, that also display labeling within nerve fibers (Drescher et al., 2010). In addition, the zebrafish inner ear expresses transcripts that encode both D1b and multiple D2R family members, suggesting that dopamine (DA) signaling may involve other cellular targets, as reported for the trout saccule (Drescher et al., 2010).

It has been suggested that in the LL DA is released in a paracrine fashion and acts at ribbon synapses, likely enhancing the activity of presynaptic Cav1.3a channels and thereby increasing neurotransmission (Toro et al., 2015). Although it has been shown that activity in LL DA neurons correlates with motor activity during fictive swimming (Jay et al., 2015), the biological function of dopaminergic modulation on HC activity is not clear. It has been suggested that such modulation may lower the threshold of response to stimulation, thereby “sensitizing” the system and thus enhancing capture of prey or the ability to avoid predators. It would be interesting to know whether the activation of DELL neurons during locomotion leads to changes in afferent activity and thus if DELL neurons respond to sensory stimuli in the absence of motor outputs.

On the other hand, the cholinergic efferent system has been hypothesized to serve many purposes: as a “feedforward” system that anticipates self-generated sensory stimulation during locomotion and inhibits the response to such stimulation, and as a “feedback” inhibitory device that reduces the response to constant excitation, protecting the system from the cytotoxic effects due to overstimulation. Early studies demonstrated that cholinergic efferent innervation suppresses afferent neuron activity during self-generated movements (Russell, 1968, 1971a; Roberts and Russell, 1972; Russell and Roberts, 1974). During swimming, cholinergic efferents are synchronously active with spinal motor neurons, leading to a reduction in spontaneous afferent activity (Lunsford et al., 2019; Pichler and Lagnado, 2020). Moreover, efference copy signal most closely translate the swim duration (Lunsford et al., 2019) and the strength of the motor signal (Pichler and Lagnado, 2020), demonstrating that efferent fibers are poised to suppress self-generated activity. However, the mechanisms involved in this phenomenon remained unsolved until Pichler and Lagnado (2020) showed that efference copy signal modulates neuromast output. These authors leveraged a fluorescent reporter of glutamate concentration to measure the spontaneous synaptic output of HCs, and performed *in vivo* Ca^2+^ imaging of efferent fiber activity while simultaneously recording from the motor nerve during fictive swimming. Notably, each fictive swimming bout was highly correlated with suppression of HC glutamate release, with corresponding reduction of afferent neuron activity. Moreover, coincidental mechanical stimulation of HCs and motor nerve activity suppressed HC synapses to a higher degree. These findings support a role for HCs as a cellular target by which efference copy signals suppress afferent neuron activity, aiding the animal to distinguish between external and self-generated stimuli. Interestingly, efferent modulation is biased toward HCs activated by posterior deflections, thus supporting the idea that the LL allows the detection of predators during swimming (Pichler and Lagnado, 2020). Since changes in motor commands don’t correlate with efferent inhibitory effect, it has been suggested that the cholinergic efferent system acts as a corollary discharge with limited temporal information from higher order motor units rather than an efference copy that encodes the timing from local motor units (Lunsford et al., 2019).

On the other hand, Mensinger et al. (2019) chronically implanted microwire electrodes into the ALL nerve of oyster toadfish and neural activity was monitored during forward free swim. Strikingly, efferent modulation was not detected and appeared unnecessary for the fish to detect outside stimuli during movement. This discrepancy opens several new avenues of study. Are HCs tuned to anterior deflections, up-regulated to further increase sensitivity to external stimulation with ensuing consequences for afferent neuron activity? Is free swimming imposing additional stimuli that could modulate the strength of the efference copy signal? Future studies are needed to examine the physiological relationship between anteriorly polarized HCs and their corresponding afferent neuron partners.

The precise connectivity patterns of HCs and efferent neurites within a neuromast started to be deciphered by Dow et al. (2018) who showed that only one efferent terminal contacts every mature HC, irrespectively of its polarity (Figure 2B). Consistent with this finding, it has been shown that during fictive locomotion presynaptic activity across all efferent synapses within a neuromast are synchronously activated (Pichler and Lagnado, 2020). Further ultrastructural studies are needed to determine if OEN or DELL neurons target afferent neurons and if axo-axonic connections between OEN and DELL neurons are established.

## The Lateral Line Efferent Cholinergic Synapse

Lateral line efferent pathway share structural and functional similarities with those of the cochlea (Table 1). Efferent stimulation to the LL leads to inhibition of afferent transmission (Russell, 1971b; Russell and Roberts, 1972; Flock and Russell, 1976; Lunsford et al., 2019; Pichler and Lagnado, 2020) and hyperpolarizing inhibitory postsynaptic potentials in HCs (Flock and Russell, 1973b). This is brought about by cholinergic efferent fibers (Dawkins et al., 2005; Zhang et al., 2018) directly contacting the base of LL HCs (Dow et al., 2018), similar to what has been described for MOC efferents (Figure 2B).

**TABLE 1:** Commonalities among MOC-OHC, mammalian vestibular efferent-type II HC and LL efferent cholinergic synapses.

	**MOC-OHC synapse**	**Mammalian vestibular efferent- type II HC synapse**	**LL efferent cholinergic synapse**
ACh-mediated effect on HCs	Hyperpolarization	Hyperpolarization	Hyperpolarization
nAChR	α9α10	α9*	α9
nAChR functionally couple to SK channels	Yes	Yes	Yes
Postsynaptic cistern	Yes	Yes	Yes
Efferent fibers make direct contact to HCs with afferent ribbon synapses	Yes^†^	Yes	Yes

*^†^(Hashimoto and Kimura, 1988; Simmons and Liberman, 1988; Liberman et al., 1990; Weisz et al., 2012).*

In the cochlea, the effects of ACh are mediated by an atypical nAChR located at the synapse between efferent fibers and OHCs. The activation of α9α10 nAChRs leads to an increase in intracellular Ca^2+^ and the subsequent opening of small conductance Ca^2+^-activated K^+^ (SK2) channels, thus leading to hyperpolarization of OHCs and reduction of electromotility (Housley and Ashmore, 1991; Fuchs and Murrow, 1992a, b; Doi and Ohmori, 1993; Elgoyhen et al., 1994; Blanchet et al., 1996; Fuchs, 1996; Nenov et al., 1996; Dulon et al., 1998; Oliver et al., 2000). As discussed in the following sections, in the LL, HC hyperpolarization is the result of the influx of cations (Na^+^ and Ca^2+^) through homomeric α9 nAChRs and the subsequent activation of Ca^2+^-sensitive SK potassium channels (Carpaneto Freixas et al., 2021).

One of the functional peculiarities of the inner ear efferent-OHC synapse is associated with an uncommon synaptic structure; the synaptic cistern, that is found within 20 nm from the plasma membrane and is co-extensive with the efferent synaptic contact (Gulley and Reese, 1977; Hirokawa, 1978; Saito, 1983; Fuchs et al., 2014). This endoplasmic organelle was described in early electron micrographs (Engstrom and Wersall, 1958; Smith and Sjöstrand, 1961; Saito, 1980) and appears to be an obligatory component of cholinergic synapses on all types of HCs. It has been proposed that the adjoining synaptic cistern acts as a tightly coupled Ca^2+^ source to serve Ca^2+^-induced Ca^2+^ release, similar to that produced by ryanodine receptors of the sarcoplasmic reticulum in skeletal muscles (Lioudyno et al., 2004). Moreover, in developing IHCs (Moglie et al., 2018; Zachary et al., 2018) and in OHCs (Moglie et al., 2021) subsynaptic cisterns provide efficient compartmentalization and tight control of cholinergic Ca^2+^ signals. Similar to cochlear OHCs, LL HCs have a postsynaptic cistern opposed to efferent terminals (Dow et al., 2018), proposed to participate in Ca^2+^ compartmentalization and/or Ca^2+^ induced Ca^2+^ release mechanisms.

Notably, the efferent-type II HC synapse found in the mammalian vestibular system also share some commonalities with the fish LL efferent cholinergic synapse (Table 1). Similar to LL HCs, vestibular type II HCs are innervated by bouton-like afferent terminals (Goldberg, 2000) and also receive direct efferent cholinergic contacts (Hilding and Wersäll, 1962; Iurato et al., 1972; Lysakowski and Goldberg, 1997). Like in the cochlea and the LL, stimulation of inner ear cholinergic efferents results in type II HCs hyperpolarization, and the effects of ACh are mediated by α9-containing (α9^∗^) nAChRs functionally couple to SK channels (Poppi et al., 2018; Yu et al., 2020). Moreover, efferent-type II HC synapses are characterized by a postsynaptic cistern structure, similar to what has been observed at efferent contacts in the cochlea and LL (Lysakowski and Goldberg, 1997). It is not yet clear whether the subsynaptic cistern and its associated pathways, as described in cochlear HCs, are similar in vestibular HCs.

### The Nicotinic Receptor at the Lateral Line Efferent Synapse

Efferent innervation mediated by α9^∗^ nAChRs is a common feature to all vertebrate HCs (Elgoyhen et al., 1994; Glowatzki and Fuchs, 2000; Hiel et al., 2000; Holt et al., 2003; Parks et al., 2017). In mammals, the best studied efferent-HC synapse, MOC efferent activity is mediated by a pentameric α9α10 nAChR, with very peculiar functional properties and a high Ca^2+^ permeability (Elgoyhen et al., 2001; Lustig et al., 2001; Sgard et al., 2002; Gómez-Casati et al., 2005; Elgoyhen and Katz, 2012). Moreover, the α10 subunit is an essential component of the HC nAChR, since in α10 knockout mice, α9 nAChRs expressed by OHC are unable to transduce efferent signals *in vivo* (Vetter et al., 2007).

Due to the structural and functional commonalities between LL and cochlear efferent synapses, it has been suggested that the nAChR at the LL efferent synapse might be composed of α9 and α10 nAChR subunits. Although similar molecules are probably universally expressed in all vertebrate efferent synapses, the mammalian α9α10 nAChR has been under positive selection, rendering a receptor with differential functional properties (Franchini and Elgoyhen, 2006; Lipovsek et al., 2012; Marcovich et al., 2020). For example, in contrast to mammalian α10 (Elgoyhen et al., 2001), chicken and *X. tropicalis* α10 subunits form a functional homomeric receptor (Marcovich et al., 2020), expanding the possibilities of combinatorial assemblies leading to receptors with distinct properties. Therefore, what has been described for mammalian efferent-HC synapses might not necessarily apply to the piscine efferent-HC synapse. The nature of the piscine cholinergic HC receptor remained unsolved until Carpaneto Freixas et al. (2021) provided strong evidences suggesting that an α9 homomeric receptor mediates synaptic transmission between efferent fibers and HCs of the zebrafish LL. In that study we analyzed single-cell RNA-seq and microarray studies in zebrafish HCs (Steiner et al., 2014; Erickson and Nicolson, 2015; Matern et al., 2018; Lush et al., 2019) and surprisingly found enriched expression of α9 but not α10 subunits. In addition, *in situ* hybridization data indicated expression of α9 mRNA, but not α10 transcripts, in LL neuromast and the posterior macula in the otic vesicle. Furthermore, we showed that zebrafish α9 nAChRs expressed in *Xenopus* oocytes are functional and exhibit robust ACh-evoked currents which are not boosted in magnitude when co-expressed with α10. This contrasts sharply with that reported for rat α9 receptors which exhibit very small ACh-evoked responses, that are non-reliable nor reproducible, and are boosted when co-expressed with α10 (Elgoyhen et al., 1994, 2001; Sgard et al., 2002). Moreover, the EC_50_ for ACh of zebrafish α9α10 nAChRs is 40-times higher than that of α9 homomeric receptors and near 500 μM, a value that is too high compared to any other known α9^∗^ nAChR EC_50_ (Elgoyhen et al., 2001; Lipovsek et al., 2012; Marcovich et al., 2020).

Biophysical and pharmacological characterization of zebrafish recombinant α9 and α9α10 nAChRs revealed that these receptors share some properties with their mammalian counterparts: they are reversibly blocked by α-bungarotoxin and strychnine, and exhibit a significant Ca^2+^ contribution to ACh-evoked responses. On the other hand, the zebrafish α9 nAChR exhibits a high desensitization rate and lacks of modulation by external Ca^2+^ (Carpaneto Freixas et al., 2021) thus differing from rat (Elgoyhen et al., 1994; Katz et al., 2000) and chicken (Lipovsek et al., 2012) α9 receptors, which exhibit low desensitization kinetics, and in the case of rat receptors (not reported for chicken), are blocked by extracellular Ca^2+^. These results are in line with the observation that within the nAChR family, α9 and α10 subunits are the ones that exhibit the highest degree of coding sequence divergence, mirrored by a great variability of functional properties across species (Franchini and Elgoyhen, 2006; Lipovsek et al., 2012; Marcovich et al., 2020).

In adult mammalian auditory epithelia, where phonoreception and sound amplification are segregated, efferent innervation targets OHC. In contrast, in birds, fish and amphibian’s efferent innervation coexists with afferent innervation in the same HC. Thus, Ca^2+^ entry could result in efferent triggered activation of afferent fibers due to Ca^2+^ spill over. In this scenario, the high desensitization kinetics of zebrafish α9 nAChRs could be key to limit the extent of Ca^2+^ influx avoiding a cross talk between efferent and afferent systems. Furthermore, other mechanisms underlying distinct excitatory and inhibitory Ca^2+^ signals within HCs might take place. During a critical developmental period when cochlear IHC are innervated by both afferent and efferent fibers, high intracellular Ca^2+^ buffering and subsynaptic cisterns provide efficient control of cholinergic Ca^2+^ signals, preserving the inhibitory signature of the cholinergic input (Moglie et al., 2018). Although it is still unknown if intracellular Ca^2+^ buffering systems are operating in LL HCs, there is evidence for postsynaptic cisterns opposed to efferent terminals (Dow et al., 2018). These findings support the idea that in zebrafish LL HCs, postsynaptic cisterns could provide efficient compartmentalization of cholinergic Ca^2+^ signals to prevent efferent-to-afferent synaptic cross-talk.

Taken together these results indicate that, different from the hearing organ of vertebrate species, an homomeric α9 nAChR operates at the LL efferent synapse. The generation of genetically modified zebrafish models will enable further understanding of the function of α9 and α10 subunits at the LL efferent-HC synapse. In particular, the analysis of knockout fish for *CHRNA9* and *CHRNA10* will provide a clear demonstration of the subunit composition of the receptors underlying ACh-mediated inhibition in the LL.

### The Inhibitory Signature of the Lateral Line Cholinergic Efferent Synapse

Within the mammalian inner ear the net effect of MOC efferent cholinergic activity is to attenuate the firing activity (both spontaneous and sound-evoked) of the auditory nerve fibers (Guinan and Gifford, 1988), presumably by reducing basilar membrane motion due to OHC hyperpolarization (Guinan and Stankovic, 1996). The activation of α9α10 nAChRs leads to an increase in intracellular Ca^2+^ and the subsequent opening of small conductance Ca^2+^-activated K^+^ (SK2) channels, thus driving HC hyperpolarization (Dulon et al., 1998).

Pioneer works in the LL of *Xenopus*, burbot *Lota lota*, and dogfish *Scyliorhynus* have shown that stimulation of cholinergic efferents, inhibits spontaneous and evoked activity of afferents by generating inhibitory postsynaptic potentials in HCs (Russell, 1971a; Russell and Roberts, 1972; Flock and Russell, 1976). In addition, recent work has demonstrated that activation of zebrafish LL cholinergic efferents suppresses glutamate release from HC (Pichler and Lagnado, 2020) and inhibits afferent activity (Lunsford et al., 2019). Altogether, these evidences suggest that the LL efferent cholinergic synapse might exhibit an inhibitory signature, similar to the MOC-OHC synapse.

**FIGURE 3:**
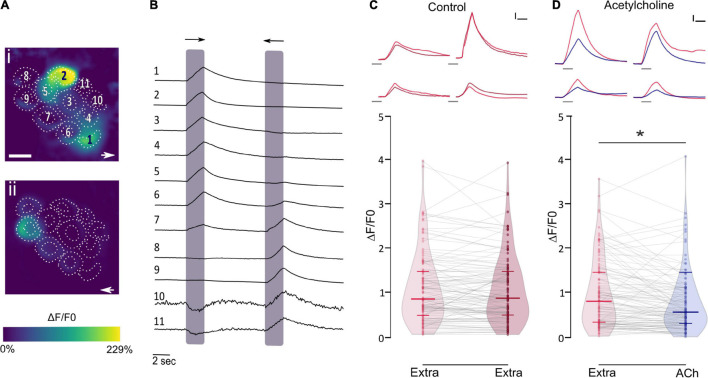
ACh inhibits mechanically evoked Ca^2+^ signals. **(A)** Representative functional Ca^2+^ images of a double-transgenic neuromast expressing GcAMP7a in HCs during a 2 s mechanical stimulus in either the anterior–posterior (i) or in the posterior–anterior direction (ii). ROIs are drawn around each HC. **(B)** Representative temporal curves of mechanosensitive Ca^2+^ responses (ΔF/F0) of HCs numbered in A. Shaded areas indicate the time when the neuromast was mechanically stimulated. **(C)** Top, Representative temporal ΔF/F0 curves of four HCs over two trials with the same stimulation 1 min apart (1° stimulus, light red; 2° stimulus, dark red). Bottom, Peak ΔF/F0 for single HCs over two trials with the same stimulation after 1 min. **(D)** Top, Representative temporal ΔF/F0 curves of four HCs before (red) and after (blue) the application of 1 mM ACh. Bottom, ACh application reduces mechanosensitive Ca^2+^ responses (*n* = 114, W = -3493, *p = 7.89e-07, MPRBC = 0.532, Wilcoxon matched-pairs signed-rank test). Calibration: **(C,D)**, 1.5 s; **(C,D)**, 25% ΔF/F0. The duration of the stimulus in **(C,D)**, top, is indicated by gray lines below each trace. Lines inside violin plots indicate the median and IQR (adapted from Carpaneto Freixas et al., 2021).

Recently this puzzle was solved and the physiological signature of the LL efferent cholinergic synapse has been characterized. Performing *in vivo* Ca^2+^ imaging on mechanically stimulated zebrafish LL HCs, we showed that ACh elicits a decrease in evoked Ca^2+^ signals (Figure 3; Carpaneto Freixas et al., 2021). Since the increase in intracellular Ca^2+^ upon deflection of the cilia results from Ca^2+^ influx through mechanosensitive ion channels (Corey and Hudspeth, 1979; Fettiplace, 2009; Zhang et al., 2018) and the subsequent activation of voltage-gated Ca^2+^ channels due to HC depolarization (Moser and Beutner, 2000; Sheets et al., 2017; Zhang et al., 2018), ACh inhibition of Ca^2+^ influx is the result of a net hyperpolarization of LL HC. We further showed that ACh-mediated effects are blocked by both α-bungarotoxin and apamin, supporting the notion that the inhibitory signature of the LL efferent cholinergic synapse is most likely served by α9^∗^ nAChRs and the subsequent activation of Ca^2+^-dependent SK potassium channels (Carpaneto Freixas et al., 2021). These findings support the generally held hypothesis that Ca^2+^ entering through the efferent nAChR activates nearby SK channels leading to HC hyperpolarization (Doi and Ohmori, 1993; Blanchet et al., 1996; Nenov et al., 1996; Yuhas and Fuchs, 1999; Glowatzki and Fuchs, 2000; Oliver et al., 2000; Holt et al., 2003; Katz et al., 2004; Dawkins et al., 2005; Gómez-Casati et al., 2005; Parks et al., 2017). In birds (Matthews et al., 2005) and mammals (Dulon et al., 1998; Oliver et al., 2000) SK2 channels are functionally coupled to α9^∗^ nAChRs. However, zebrafish HC express not only SK2 but also SK1 transcripts (Cabo et al., 2013; Carpaneto Freixas et al., 2021). Since SK1 and SK2 are generally co-expressed in the brain of the electric fish *Apteronotus leptorhynchus* (Ellis et al., 2008) and mammals (Stocker, 2004), and given that rat SK1 forms functional channels with SK2 (Benton et al., 2003; Autuori et al., 2019), it could be proposed that heteromeric SK1/SK2 channels might be functionally coupled to α9^∗^ nAChRs. However, the fact that apamin blocks ACh-mediated effects suggests that SK2 channels and not SK1 play a key role in zebrafish LL HC hyperpolarization, since SK2 channels are the most apamin-sensitive (Köhler et al., 1996; Shah and Haylett, 2000; Strøbæk et al., 2000; Stocker, 2004). Therefore, additional studies are needed to determine the role of SK1 channels in fish LL organ.

Outer hair cells in the higher frequency regions of the mammalian cochlea express Ca^2+^-sensitive BK potassium channels, which are functionally coupled to α9α10 nAChRs and contribute directly in the synaptic (efferent) inhibition of OHCs (Wersinger et al., 2010; Maison et al., 2013; Rohmann et al., 2015). In teleost fish, pharmacological block of BK channels (Rohmann et al., 2013) and knockdown of *slo1a* and *slo1b*, duplicate genes that code for the α-subunit of BK channels (Rohmann et al., 2009), results in increased auditory thresholds (Rohmann et al., 2014), thus suggesting that BK channels regulate peripheral hearing sensitivity among fishes. In addition, scRNA-Seq of zebrafish LL HC revealed that within a neuromast mature HC differentially express the gene that codes for the α-subunit of BK channels (Lush et al., 2019). Since LL α9^∗^ nAChRs are highly permeable to Ca^2+^ (Carpaneto Freixas et al., 2021), influx through these channels might raise Ca^2+^ to levels sufficient to activate BK channels. It will be of interest to learn if, like in the higher frequency regions of the mammalian cochlea, BK channels also contribute to ACh inhibition of LL HCs.

Interestingly, we found that the ACh-mediated effect on HCs is heterogeneous and independent of their polarity (Carpaneto Freixas et al., 2021). Functional heterogeneity among HCs within the same neuromast has been shown previously, since stimuli able to open mechanosensitive channels are insufficient to evoke vesicle fusion in the majority of HCs (Zhang et al., 2018). Moreover, synaptically active HCs exhibit lower intracellular K^+^ levels than silent HCs. In addition, physiological heterogeneity has also been shown for LL afferent response to efferent activity (Lunsford et al., 2019). Differences in the density of α9^∗^ nAChRs mediating Ca^2+^ influx and/or SK channels causing hyperpolarization could explain this phenomenon, and further studies are needed to shed light on this issue.

The finding that the ACh-mediated effect is independent of HC polarity is in accordance with ultrastructural data showing that within a neuromast OEN efferent fibers do not form polarity-specific connections with HCs (Dow et al., 2018). Moreover, during fictive locomotion presynaptic activity across all efferent synapses within a neuromast are synchronously activated (Pichler and Lagnado, 2020). However, Pichler and Lagnado (2020) reported that efferent modulation is highly selective for HCs activated by posterior deflections, as would occur during forward motion. This discrepancy poses new questions for future studies. Do differences in the efficiency of presynaptic ACh release at efferent terminals and/or in the number of efferent terminals per HCs of different polarities account for this biased efferent modulation? Alternatively, could physiological heterogeneity of LL HCs contribute to differences in the efficiency with which depolarization triggers glutamate release?

## Conclusion

**FIGURE 4:**
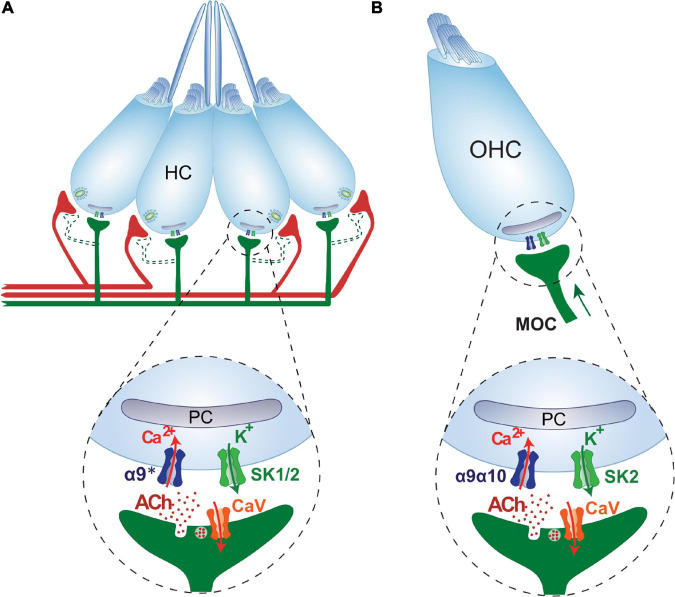
Cholinergic lateral line and cochlear efferent synapses share structural, functional and molecular similarities. **(A)** Schematics of the cholinergic LL efferent synapse. LL HCs are innervated by afferent (red) and cholinergic efferent (green) fibers. Evidence for efferent cholinergic fibers contacting afferent neurons (dashed light green) is still missing. The net effect of LL efferent cholinergic activity is to hyperpolarize HCs. This is mediated by the activation of an α9* nAChR with high Ca^2+^ permeability. Subsequent activation of Ca^2+^-dependent K^+^ SK channels drives HC hyperpolarization (Carpaneto Freixas et al., 2021). Postsynaptic cisterns (PC) opposed to efferent terminals are co-extensive with the efferent synaptic contact (Dow et al., 2018). **(B)** Schematics of the cholinergic MOC-OHC efferent synapse. OHCs are innervated by MOC cholinergic efferent (green) fibers. The net effect of MOC activity is to hyperpolarize OHCs. This is mediated by the activation of an α9α10 nAChR with high Ca^2+^ permeability (Elgoyhen et al., 2001; Lustig et al., 2001; Sgard et al., 2002; Gómez-Casati et al., 2005; Elgoyhen and Katz, 2012). Subsequent activation of Ca^2+^-dependent K^+^ SK2 channels drives OHC hyperpolarization (Dulon et al., 1998). Postsynaptic cisterns (PC) opposed to MOC terminals have been proposed to participate in Ca^2+^ compartmentalization and/or Ca^2+^-induced Ca^2+^ release mechanisms.

Due to the overall similarity between mammalian and piscine LL efferent synapses (Figure 4), zebrafish emerges as an excellent platform to study auditory disorders and evaluate compounds that target α9^∗^ nAChRs to treat pathologies related to the auditory pathway. Recent works showed a positive correlation between the degree of hidden hearing loss (HHL) prevention and the level of MOC activity (Boero et al., 2018) and provided evidence that olivocochlear-mediated resistance to presbycusis occurs via the α9α10 nAChR on OHCs (Boero et al., 2020). These findings highlight the potential use of drugs that increase α9α10 nAChR activity as a pharmacotherapeutic strategy to avoid HHL and prevent presbycusis. In addition, the transient MOC innervation to the IHCs provides a tight regulation of prehearing spontaneous activity, and is crucial for the development of the central auditory pathway (Clause et al., 2014; Di Guilmi et al., 2019).

One significant advantage of studying HC and their synapses in zebrafish is that LL neuromasts are optically and pharmacologically accessible, enabling the study of HC *in vivo* and in their native environment. Moreover, zebrafish is amenable to rapid transgenic modification to express tissue specific transgenes encoding fluorescent markers or gene products (Kwan et al., 2007). This is especially useful in the transparent larvae where HC structure can easily be visualized *in vivo* and dynamic cellular processes can be imaged in a live, intact preparation (Esterberg et al., 2014; Graydon et al., 2017; Pickett et al., 2018; Zhang et al., 2018; Wong et al., 2019; Holmgren and Sheets, 2021; Sheets et al., 2021).

Deciphering the molecular players at the zebrafish cholinergic LL efferent synapse will enable the generation of molecular tools to selectively manipulate its activity and evaluate its role on several processes such as sensory processing, HC death in response to ototoxic drugs, HC regeneration, assembly of the auditory circuit and noise-induced hearing loss. The advances made from those studies could contribute to the understanding of auditory disorders and will aid in developing preventive or protective therapies in the future.

## Author Contributions

PP designed the review and wrote the first draft. AE contributed to the final version of the review. Both authors approved the submitted version.

## Conflict of Interest

The authors declare that the research was conducted in the absence of any commercial or financial relationships that could be construed as a potential conflict of interest.

## Publisher’s Note

All claims expressed in this article are solely those of the authors and do not necessarily represent those of their affiliated organizations, or those of the publisher, the editors and the reviewers. Any product that may be evaluated in this article, or claim that may be made by its manufacturer, is not guaranteed or endorsed by the publisher.
